# 695. Antipseudomonal Versus Narrow-spectrum Agents for the Treatment of Community-onset Intra-abdominal Infections

**DOI:** 10.1093/ofid/ofab466.892

**Published:** 2021-12-04

**Authors:** Lacy Worden, Lisa E Dumkow, Lisa E Dumkow, Kali VanLangan, Thomas Beuschel, Andrew Jameson

**Affiliations:** 1 Mercy Health Saint Mary's, Grand Rapids, Michigan; 2 Ferris State University, Grand Rapids, Michigan

## Abstract

**Background:**

Antipseudomonal antibiotic regiments are often used to treat community-acquired intra-abdominal infections (CA-IAI) despite common causative pathogens being susceptible to more narrow-spectrum agents. The purpose of this study was to compare post-infection complications in adult patients treated for CA-IAI with antipseudomonal or narrow-spectrum regimens

**Methods:**

This retrospective cohort study included patients ≥18 years admitted for CA-IAI treated with antibiotics between January 1, 2013, and December 31, 2019. Patients who had bacteremia or peritonitis were excluded. The primary objective of this study was to compare post-infection complications within 90 days between patients treated empirically with antipseudomonal versus narrow-spectrum regimens. Post-infection complication was defined as post-operative infection, recurrence of diverticulitis, or mortality. Secondary objectives were to compare infection and treatment characteristics along with patient outcomes. Sub-group analyses were planned to compare outcomes of patients with low-risk and high-risk CA-IAI and patients who required surgical intervention versus who were medically managed

**Results:**

A total of 350 patients were included: Antipseudomonal, n=204; Narrow-spectrum, n=146. There were no differences in 90-day post-infection complications between groups (Antipseudomonal 15.1% vs Narrow-spectrum 11.3%, p=0.296). Additionally, no differences were observed in hospital LOS, 90-day readmission, *C. difficile*, or mortality. Patients treated with Antipseudomonal regimens received longer durations of therapy (median 11 days [IQR 8-14] vs 9 days [IQR 5-12], p< 0.001). No differences were observed in 90-day post-infection complications for patient with low-risk (Antipseudomonal 15% vs Narrow-spectrum 9.6%, p=0.154) or high-risk CA-IAI (Antipseudomonal 15.8% vs Narrow-spectrum 22.2%, p=0.588), or those who were surgically (Antipseudomonal 8.5% vs Narrow-spectrum 9.2%, p=0.877) or medically managed (Antipseudomonal 17.5% vs Narrow-spectrum 13.1%, p=0.463).

**Conclusion:**

Post-infection complication rates were similar among patients treated with antipseudomonal and narrow-spectrum antibiotics. Antipseudomonal therapy is likely unnecessary for most patients with CA-IAI

**Disclosures:**

**Lisa E. Dumkow, PharmD, BCIDP**, Nothing to disclose

